# The Biased G-Protein-Coupled Receptor Agonism Bridges the Gap between the Insulin Receptor and the Metabolic Syndrome

**DOI:** 10.3390/ijms19020575

**Published:** 2018-02-17

**Authors:** Iryna Liauchonak, Fady Dawoud, Yatin Riat, Bessi Qorri, Manpreet Sambi, Justin Jain, Regina-Veronicka Kalaydina, Nicole Mendonza, Komal Bajwa, Myron R. Szewczuk

**Affiliations:** 1 Postgraduate Medical Education, Graduate Diploma and Professional Master in Medical Sciences, School of Medicine, Queen’s University, Kingston, ON K7L 3L4, Canada; iryna.liauchonak@gmail.com (I.L.); fady.dawoud@hotmail.com (F.D.); riatyatin@gmail.com (Y.R.); justin.jain@queensu.ca (J.J.); bajwa.komal@queensu.ca (K.B.); 2 Department of Biomedical and Molecular Science, Queen’s University, Kingston, ON K7L 3N6, Canada; bessi.qorri@queensu.ca (B.Q.); m.sambi@queensu.ca (M.S.); nicka.kalaydina@queensu.ca (R.-V.K.); nicole.mendonza@queensu.ca (N.M.)

**Keywords:** insulin receptor signaling, G protein-coupled receptors, metabolic syndrome, GPCR biased agonism, angiotensin, bradykinin, gut microbiota, ghrelin, polycystic ovarian syndrome, pancreatic cancer, Alzheimer’s

## Abstract

Insulin signaling, as mediated through the insulin receptor (IR), plays a critical role in metabolism. Aberrations in this signaling cascade lead to several pathologies, the majority of which are classified under the umbrella term “metabolic syndrome”. Although many of these pathologies are associated with insulin resistance, the exact mechanisms are not well understood. One area of current interest is the possibility of G-protein-coupled receptors (GPCRs) influencing or regulating IR signaling. This concept is particularly significant, because GPCRs have been shown to participate in cross-talk with the IR. More importantly, GPCR signaling has also been shown to preferentially regulate specific downstream signaling targets through GPCR agonist bias. A novel study recently demonstrated that this GPCR-biased agonism influences the activity of the IR without the presence of insulin. Although GPCR-IR cross-talk has previously been established, the notion that GPCRs can regulate the activation of the IR is particularly significant in relation to metabolic syndrome and other pathologies that develop as a result of alterations in IR signaling. As such, we aim to provide an overview of the physiological and pathophysiological roles of the IR within metabolic syndrome and its related pathologies, including cardiovascular health, gut microflora composition, gastrointestinal tract functioning, polycystic ovarian syndrome, pancreatic cancer, and neurodegenerative disorders. Furthermore, we propose that the GPCR-biased agonism may perhaps mediate some of the downstream signaling effects that further exacerbate these diseases for which the mechanisms are currently not well understood.

## 1. Introduction

Activation of the insulin receptor (IR) and its downstream signaling is critical to maintaining metabolic homeostasis and represents a significant area of interest in biology and medicine [1]. This receptor is of particular relevance due to its crucial role in metabolism and the development of metabolic syndrome. Metabolic syndrome (MetSyn) is defined as the combination of conditions that increase the risk of cardiovascular disease and type two diabetes mellitus (T2DM) [2]. However, the specific underlying biochemical and cellular properties of IR signaling that may contribute to the development of this syndrome have not yet been identified.

IR activation follows the conventional ligand-receptor interaction and involves the binding of insulin to the IR, resulting in broad signaling that occurs through three main biochemical steps. First, tyrosine phosphorylation of the IR and its substrates occurs and is followed by the activation of phosphoinositide 3-kinase (PI3K) (a lipid kinase), and culminates with the activation of serine/threonine kinases, the most important being protein kinase B (AKT) [3]. Although it is still debated, it has been well-documented that activation of the IR requires ligand-induced receptor dimerization, which is a characteristic of the entire family of receptor tyrosine kinases (RTKs) and also a ubiquitous property of G-protein-coupled receptors (GPCRs) [4]. Although IR and GPCR signaling initiate separate signaling cascades exclusive to these receptors, a growing body of evidence has identified a previously unknown link between the two signaling platforms. Evidence indicating a “missing link” suggests that the IR initiates metabolic events by signaling through a heterotrimeric G protein [1,5].

Although GPCRs are some of the most comprehensively studied and well-characterized proteins, recent studies have revealed an additional facet of these proteins, namely the phenomenon of biased agonism or functional selectivity, which is characterized as preferential activation of certain pathways by a ligand [6,7]. The novel concept of the GPCR-biased agonism has been further developed by the recent discovery of a bias that results in IR activation and signaling in the absence of insulin [8]. This unique finding may represent the missing link between GPCR and IR signaling that could contribute to the development of the complex conditions of metabolic syndrome. Here, we aim to provide an overview of GPCR-biased agonism and its proposed mechanism of action in activating the insulin receptor in the absence of insulin. We also aim to highlight specific ligands that promote this bias and the critical roles they play in metabolic syndrome. Finally, we aim to expand on several pathologies that are either classified as or are exacerbated by metabolic syndrome and identify additional disease states on which GPCR-biased agonism for the insulin receptor may play a role.

## 2. Mechanism of Action

### 2.1. An Overview of Normal Insulin Receptor Signaling

The insulin receptor is synthesized as a preproreceptor. Following cleavage of a signal peptide, the proreceptor undergoes glycosylation, folding, and dimerization [9,10]. In a similar fashion to other RTKs, the final IR consists of a heterotetrameric complex comprised of two extracellular α subunits (IRα) and two membrane-spanning tyrosine kinase-containing β subunits (IRβ) linked by disulfide bonds [10]. Following insulin binding to the IRα, a conformational change occurs in the receptor that transmits a signal across the plasma membrane and activates the intrinsic tyrosine kinase domain within the IRβ. This change results in a series of intermolecular auto-phosphorylation reactions on the tyrosine residues, at which point the IR recruits various substrates and proteins to exert its downstream effects [9].

**Figure 1:**
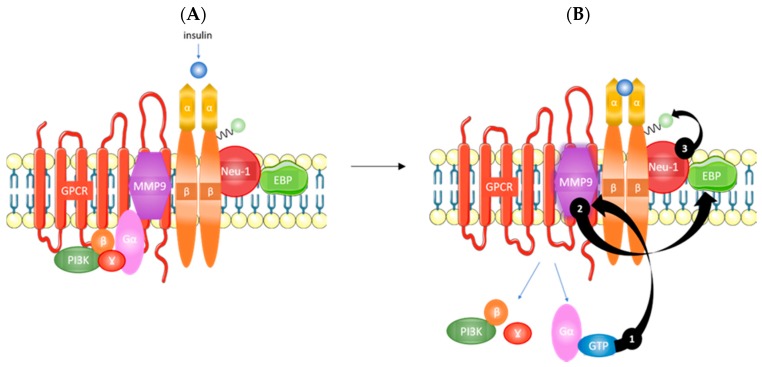
Overview of insulin signaling. (**A**) Insulin binding to the IRα and (**B**) potentiates GPCR signaling that activates MMP9 (1). Active MMP9 relieves restrictive EBP that activates Neu-1 (2). Activated Neu-1 cleaves α-2,3 sialic acid residues on IRβ to remove steric hindrance and allow for receptor dimerization (3). IRα, α subunit of the insulin receptor; GPCR, G-protein coupled receptor; MMP9, matrix metalloproteinase 9; EBP, elastin binding protein; Neu-1, neuraminidase-1; IRβ, β subunit of the insulin receptor; PI3K, phosphoinositide 3-kinase. Taken in part from Alghamdi et al. [11] © 2014 The Authors. http://dx.doi.org/10.1016/j.cellsig.2014.02.015. Published by Elsevier Inc. This is an open access article under the CC BY-NC-ND license (http://creativecommons.org/licenses/by-nc-nd/3.0/).

The highly glycosylated state of the IR [10] is particularly relevant to its possible relationship with the GPCR-biased agonism. Specifically, Alghamdi and colleagues have shown that the cleavage of α-2,3 sialic acid residues on the insulin receptor, as well as other RTKs, plays a critical role in receptor activation [11]. As shown in Figure 1, the IRα subunits potentiate GPCR-signaling and matrix metalloproteinase-9 (MMP9) activation. MMP9 removes elastin-binding protein (EBP) that exists as part of a multi-enzymatic complex that contains neuraminidase-1 (Neu-1). Activated Neu-1 hydrolyzes α-2,3 sialic acid residues on the IRβ subunits to remove steric hindrance and allow for receptor dimerization. Once the phospho-IRβ subunits phosphorylate insulin receptor substrate 1 (IRS1), an intracellular insulin signaling cascade is initiated via several pathways, including the Ras-mitogen-activated protein kinase (MAPK) and PI3K-AKT pathways [11]. Therefore, this body of evidence suggests that GPCR-biased agonism could mediate the development of insulin resistance.

### 2.2. Proposed G Protein-Coupled Receptor (GPCR) Biased Agonism

**Figure 2:**
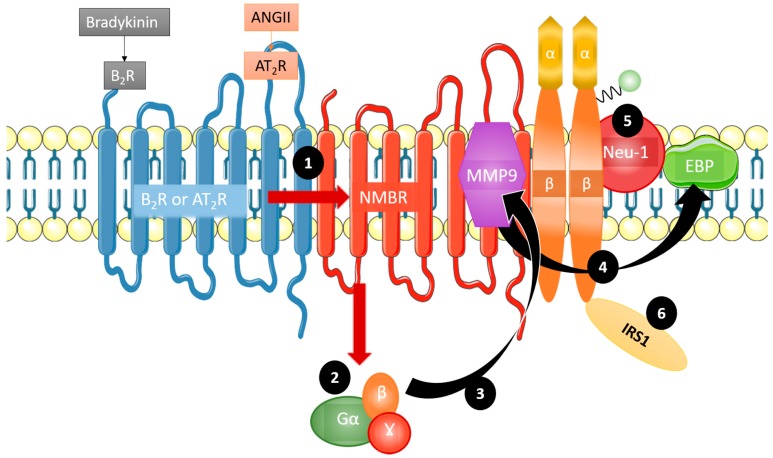
Mechanism of GPCR agonism bias towards insulin receptor activation. Bradykinin and angiotensin II are complexed with the neuromedin B receptor (NMBR), IRβ, and Neu-1. Bradykinin and angiotensin II preferentially lead to insulin receptor signaling by first forming a complex with NMBR (1). This heterodimerization leads Gα, β, and γ (2) to activate MMP-9 (3). Upon activation, MMP-9 removes EBP (4), which in turn activates Neu-1 (5). Crosstalk between these activated components leads to the phosphorylation and subsequent activation of insulin receptor substrate 1 (IRS1) (6), initiating the phosphoinositide 3-kinase - protein kinase B (PI3K-AKT) pathway, in addition to others, without the presence of insulin. Taken in part from Haxho et al [8] © 2017 The Authors © 2017 Published by Elsevier Inc. https://doi.org/10.1016/j.cellsig.2017.12.006. The CC-BY license allows users to copy, to create extracts, abstracts and new works from the Article.

As discussed, GPCRs are implicated in IR signaling [11]. Haxho et al. recently discovered an innovative approach for targeting insulin receptor signaling by exploiting GPCR-biased agonism with the neuromedin B receptor (NMBR) [8]. The finding that GPCR activation in the absence of insulin correlated with downstream IR signaling constituted the basis for this approach [12]. Additional evidence substantiating the concept of GPCR-biased agonism with NMBR for IR activation includes the discovery that NMBR exists in a complex with IRβ subunits on the cell membrane [11]. Haxho et al. found that GPCR agonists bombesin, bradykinin (BK), angiotensin I, and angiotensin II each significantly and dose-dependently induced Neu-1 sialidase activity and IR activation in human IR-expressing rat hepatoma cell lines (HTC-IR) in the absence of insulin [8]. Protein expression analyses demonstrated that the four GPCR agonists induced phosphorylation of IRS1. Additionally, it was found that angiotensin II type I receptor existed in a multimeric receptor complex with Neu-1, suggesting a molecular link for GPCR agonist-induced IR transactivation signaling mediated by Neu-1 and the modification of IR glycosylation [8] (Figure 2).

This novel GPCR-biased agonism in relation to activating the IR, irrespective of the presence of its ligand, has significant implications in MetSyn and may perhaps address the unknown aspects of disease progression, particularly as it relates to cardiovascular disease, irritable bowel disease, obesity, polycystic ovarian syndrome (PCOS), pancreatic cancer, and neuropathologies.

## 3. Metabolic Syndrome

Metabolic syndrome is defined as a combination of cardio-metabolic risk factors that lead to obesity, T2DM, and hypertension, all of which are primarily characterized by unhealthy body measurements [2,9]. The manifestation of MetSyn has been attributed to insulin resistance; as such, for this review, we will focus on insulin signaling and insulin resistance (used interchangeably with low insulin sensitivity) as it pertains to MetSyn [9]. It is thought that the peripheral effects of insulin signaling and the body’s resistance to it account for the differential expression of MetSyn observed across individuals and its associated conditions such as polycystic ovary syndrome (PCOS) [13]. Therefore, a thorough understanding of insulin resistance and the IR in pathologies that are directly and indirectly related to MetSyn is essential, particularly when identifying whether GPCR-biased agonism could contribute to the development of insulin resistance through the over-activation of IR signaling.

## 4. Metabolic Syndrome and the Relationship between Insulin Receptor (IR) in Mitigating a Pathological Phenotype

Due to metabolic syndrome being a combination of disorders that involves changes in sensitivity to insulin and by extension, the action of the IR, it is perhaps not surprising that alterations in the activity of the IR have been correlated with several pathologies including hypertension, type 2 diabetes mellitus, and obesity [2,9], in addition to cardiovascular disease and diseases of the gastrointestinal (GI) tract. As such, underlying mechanisms that contribute to these diseases may be related to the novel phenomenon of GPCR-biased agonism for IR activation. An additional facet that may contribute to this complexity is the possibility of the gut microbiota composition modulating this link.

### 4.1. Cardiovascular Disease: Implications of the Renin-Angiotensin System in IR Signaling

The renin-angiotensin system (RAS) plays a central role in the physiological and pathological responses of the cardiovascular system. Its primary effector hormone, angiotensin II (ANG II), induces its effects through GPCR signaling pathways as well as IR stimulation [14]. Both signaling pathways have also been shown to converge together in order to mediate their effects [15].

The majority of the physiological effects of ANG II, including vasoconstriction and blood pressure regulation, are mediated by angiotensin type 1 receptors (AT_1_Rs). AT_1_Rs are part of the GPCR superfamily and are widely distributed in all organs including the liver, adrenal glands, brain, lungs, kidneys, heart, and vasculature [16]. The ANG II GPCR-dependent pathway that contributes to vasoconstriction has been extensively researched and explained in detail elsewhere [17]. In brief, AT_1_Rs form complexes that activate phospholipase C (PLC), which in turn activates inositol-1,4,5-triphosphate (IP_3_) and diacylglycerol (DAG) within seconds. IP_3_ binds to its receptor on the sarcoplasmic reticulum, allowing for the efflux of calcium into the cytoplasm. Activated myosin light chain kinase (MLCK) phosphorylates the myosin light chain and enhances the interaction between actin and myosin, resulting in smooth muscle cell contraction [18]. DAG then leads to sustained protein kinase C (PKC) activation, which promotes sustained muscle contraction, revealing the underlying role that GPCRs may play in the pathogenesis of hypertension [19].

Some clinical and pharmacological studies have confirmed the link between GPCR signaling and insulin receptor cascade activation by ANG II, in which ANG II infusion is known to induce insulin resistance, and ANG II converting enzyme inhibitors (ACE) inhibitors and AT_1_R blockers improve insulin sensitivity [20]. Moreover, the ANG II receptor antagonist, irbesartan, improved whole-body insulin sensitivity in part due to improved glucose transport in skeletal muscles. Additionally, chronic administration of irbesartan was accompanied by a reduction of fasting plasma glucose [21]. This enhanced hepatic insulin sensitivity and diminished hepatic production in the fasting state may constitute possible explanations for the observed effects of irbesartan.

**Figure 3:**
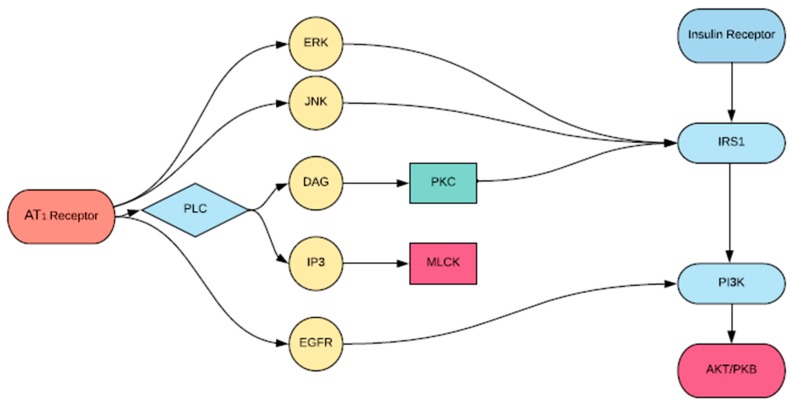
Proposed scheme of angiotensin (ANG) II-induced inhibition of protein kinase B (AKT) activation by insulin. Vascular agonist ANG II activates protein kinase C (PKC), which inhibits phosphorylation of insulin receptor substrate 1 (IRS1), phosphoinositide 3 kinase (PI3K) activation, and AKT activation.

Insulin resistance is proposed to be a significant driver of cardiovascular disease [22]. Recent reports have demonstrated that ANG II interacting with the IR can lead to insulin resistance, representing an underlying mechanism that could lead to cardiovascular disease. Specifically, ANG II interrupts IRS1 signaling at multiple levels, which may explain the severity of cardiovascular diseases in diabetic patients [14]. The ACE inhibitor, temocapril, significantly reduced plasma glucose and insulin concentration in mice with T2DM by enhancing the action of the bradykinin-nitric oxide (BK-NO) system [23]. This finding may be due to the fact that IR signaling involves the phosphatidylinositol 3-kinase (PI3K) pathway, with IRS1 binding PI3K being necessary to elicit the downstream actions of insulin [24]. For example, AKT is a downstream effector of PI3K and plays a central role in the metabolic action of insulin including glucose transport, synthesis of glycogen, and insulin-induced vasodilation [25]. The complexity of its activation is crucial to understanding the advent of insulin resistance as a result of the RAS. For example, downregulation of AKT by PKC may explain the development of insulin-resistant states. Several studies have shown that AT_1_R stimulation leads to activation of PKC [26], while others have shown that prior activation of PKC-α inhibits the ability of insulin to stimulate the enzymatic activity of AKT [27]. Figure 3 shows a schematic diagram by which these interactions occur. This GPCR-IRS1 crosstalk in the activation of PKC may explain the development of severe cardiovascular pathology in insulin-resistant patients [27].

Additionally, AT_1_R-mediated signaling leads to the activation of the extracellular signal-regulated kinase (ERK) and c-Jun N-terminal kinase (JNK) pathways [28]. Andreozzi et al. [29] demonstrated that ANG II impaired insulin signaling via ERK/JNK activation and increased phosphorylation of IRS1, resulting in a downregulation of PI3K-AKT activation, which as mentioned, is a crucial component of insulin signaling [28].

The pathogenesis of chronic vascular diseases such as those implicated in MetSyn is dependent on the complex crosstalk of various cell signaling systems. The RAS and IR signaling pathways are related through shared mediators and effectors, thereby implicating RAS activation in the development of insulin resistance within metabolic syndrome due to ANG II promoting the pathophysiology of atherosclerosis, hypertension, and congestive heart failure in endothelial cells [30]. A more thorough understanding of this connection may aid in the development of more efficacious pharmacological agents for both hypertension and insulin resistance.

### 4.2. Bradykinin, Irritable Bowel Syndrome, and Metabolic Syndrome

Metabolic regulation and inflammation are highly integrated processes contributing to metabolic syndrome, with the prothrombotic and proinflammatory properties of MetSyn having direct implications for conditions such as inflammatory bowel disease (IBD) [31,32]. One of the two major forms of IBD, ulcerative colitis (UC), is a chronic non-specific colitis involving lesions to the mucosa and submucosa [33]. The co-occurrence of MetSyn and IBD in some patients is important to consider, as the presence of both of these conditions translates to a higher risk of developing atheroembolic disease and colorectal cancer [34,35]. Despite the fact that only one study by Yorulmaz et al. reported a higher frequency of MetSyn in patients with UC [36], it is possible that a common molecular mechanism may underpin both of these conditions.

**Figure 4:**
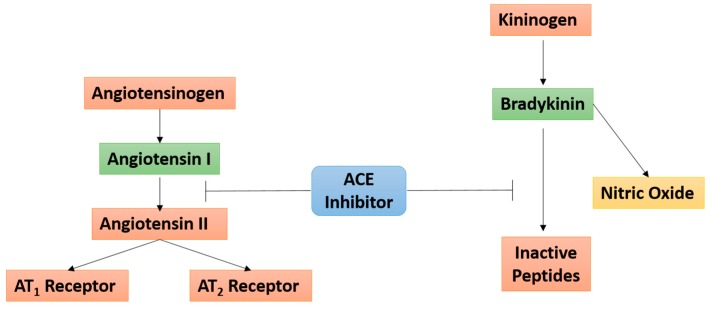
Angiotensin converting enzyme (ACE) inhibitors linking the angiotensin and bradykinin signaling pathways. ACE inhibitors prevent the conversion of angiotensin I to angiotensin II within the renin-angiotensin system, and prevent the conversion of bradykinin to inactive peptides in the kallikrein-kinin system. The T bars are indicative of the inhibitory action of ACE on activated Angiotensin I (as shown by the green box) and Bradykinin. The red boxes indicated inactive proteins.

Bradykinin (BK), a key bioactive kinin in the multienzyme cascade in the kallikrein-kinin system (KKS), has been implicated in the pathogenesis and progression of UC via the mediation of vasodilation, inflammation, pain, and edema UC [37,38,39,40,41,42]. It is believed that BK exerts its effects via high-affinity binding at the constitutively expressed bradykinin 2 receptor (B_2_R) throughout the central and peripheral tissues [33]. Moreover, while the ACE is known to play a role in BK inactivation, ACE inhibitors have been shown to enhance the effects of BK at the B_2_R [43] (Figure 4).

Due to the close relationship between the ACE and KKS systems, ACE inhibitors and B_2_R agonists such as BK have been suggested to represent new ways of targeting insulin resistance [23,44]. Shiuchi et al. showed that HOE-140, an antagonist at the B_2_R, as well as the nitric oxide (NO) synthase inhibitor, l-NAME, both attenuated the observed enhanced glucose uptake by temocapril, an ACE inhibitor [23]. More recently, Talbot and colleagues showed that B_1_R contributes to insulin resistance and metabolic syndrome while claiming that B_2_R has been associated with a preventive role in insulin resistance [44]. In addition to contributing to T2DM, BK also appears to play a direct role in UC [37,38,39,40,41,42]. BK and other selective B_2_R agonists have been shown to induce contraction in both normal healthy and inflamed mouse colon preparations, as well as in induced colitis models with upregulated B_2_R densities [40]. In a B_1_R knockout mouse model in which exposure to dextran sulfate sodium (DSS) was used to chemically induce UC, prevention of colitis exacerbation was achieved entirely with a selective B_2_R antagonist [37]. In several other DSS-induced UC models, treatment with a selective B_2_R antagonist was reported to reduce experimental colitis significantly [39,42]. Taken together, the fact that BK plays a role in both MetSyn and UC, as well as due to the co-occurrence of these conditions in certain populations, we predict that a potential molecular mechanism directly relating MetSyn and UC may exist involving GPCR-biased agonism for the IR.

Until now, there has been a lack of evidence suggesting a relationship between the insulin receptor, MetSyn, and BK in inducing UC. However, GPCR-biased agonism involving BK, the IR, and the PI3K-AKT signaling pathway may represent the missing link between UC and MetSyn [8]. Haxho et al. recently reported that B_2_R exists in a multimeric receptor complex with the NMBR, IRβ, and Neu-1 in naïve and stimulated HTC-IR cells [8]. BK signaling at the cell surface initiates a biased GPCR agonist-induced, IRβ trans-activation-signaling axis mediated by Neu-1 sialidase and glycosylation of the IR [8]. However, the imbalanced cytokine secretion and the ensuing inflammatory responses that accompany them induce mucosal damage, leading to the development of UC. Huang et al. reported that p-AKT expression was significantly increased in the intestinal mucosa of UC patients compared with controls [33]. Additionally, Hayashi et al. reported that deficiency of PI3K resulted in suppression of DSS-induced acute colitis via the production of interleukin-10 (IL-10) in intestinal macrophages [45]. Considering that both BK and the PI3K-AKT signaling pathway contribute to UC, it is possible that NMBR-MMP9-IRβ crosstalk may be one of the molecular mechanisms relating BK and PI3K-AKT signaling to the induction of UC. The findings of Haxho et al. give rise to the possibility that BK initiates the PI3K-AKT signaling via biased GPCR agonist-induced transactivation of IRβ [8].

While the application of GPCR agonists such as BK for the treatment of conditions that fall under metabolic syndrome represents immense potential, patients who suffer from both IBD and metabolic syndrome must be taken into special consideration. If GPCR agonists such as BK are deemed as therapeutic targets for MetSyn, those who are at an increased risk of developing IBD or currently living with it as comorbidity will need to be taken into account. The GPCR biased agonist BK may exacerbate IBD in patients already living with the condition, as evidenced in various in vivo and in vitro models [37,38,39,40,41,42], and consequently, alternative approaches to the management of metabolic syndrome may be warranted in this population. The link proposed to exist between BK and PI3K via biased GPCR and IR signaling [8] requires further investigation to determine whether GPCR agonists can be used in the treatment of metabolic syndrome in patients with IBD. Based on available preclinical data, it may be the case that exacerbation of IBD [41] may be an unwanted side-effect of GPCR agonist therapy in this subgroup of patients. While metabolic syndrome is serious comorbidity for patients with IBD that should be managed, the use of GPCR agonists may be ill-advised due to newfound NMBR-MMP9-IRβ crosstalk that may be contributing to UC.

### 4.3. Moderators of the IR-GPCR Signaling Link

#### 4.3.1. Gut Microbiota

The gut microbiota consists of millions of bacteria, viruses, and fungi, and begins taking shape during childbirth [46]. Although the interactions and effects of the gut flora with the human host are not fully understood, it has been implicated in the development of insulin resistance, obesity, and metabolic syndrome, and may be mediated through interactions with the IR or GPCRs [47]. For example, there are distinct differences in the major phyla that comprise the gut microbiota of lean versus obese individuals. Obese individuals have higher concentrations of *Firmicutes* than *Bacteroidetes* compared to leaner individuals who have a higher *Bacteroidetes* and lower *Firmicutes* concentration [48]. These findings suggest that the phyla of bacteria found in the human gut are correlated with the development of obesity, implicating the gut flora in insulin metabolism. The link between the IR and GPCR may result in changes in gut motility and permeability of individual molecules, which in turn get absorbed into the systemic circulation and lead to the activation of different metabolic and inflammatory processes [49].

Since it is well established that GPCRs closely interact with the insulin receptor, they are thought to affect insulin metabolism, including the amount of energy that is readily available to the host, and are involved in lipid and insulin metabolism. Samuel et al. [50] have shown that mice deficient in certain subtypes of GPCR proteins, particularly Gpr41, are significantly leaner and weigh less than control mice. Gpr41 and Gpr43 are both receptors for short chain fatty acids (SCFAs) and can be found in the colon, adipocytes, and many other tissues. They are activated by SCFAs such as acetate, propionate, and butyrate, with different SCFAs binding with various affinities to each of Gpr41 and Gpr43 [51]. Ligand stimulation of Gpr41 or Gpr43 by SCFAs releases hormones such as leptin that slow down gut motility, allowing more SCFAs to be extracted and stimulate lipogenesis [52]. Gpr43 activation has also been shown to lead to inhibition of the insulin signaling pathway in adipose tissues, which results in suppression of fat accumulation and weight loss in mice [53]. These studies suggest a strong interaction between the gut microbiota, both GPCR proteins, and the insulin receptor.

The development of a low-grade inflammatory state seen in metabolic syndrome represents another mechanism by which the gut microbiome affects insulin metabolism, described as metabolic endotoxemia [54]. Fat-rich diets alter the gut microbiome and favor the colonization of gram-negative bacteria. Lipopolysaccharides (LPS), which are released from the cell walls of dying bacteria are absorbed into the systemic circulation and are thought to contribute to metabolic endotoxemia [55]. Once LPS binds CD14 proteins on the surface of macrophages, multiple inflammatory processes are initiated that ultimately affect insulin sensitivity. Cani et al. reported that mice experiencing inflammation had increased weight, hyperglycemia, and hyperinsulinemia while mice with mutant CD14 proteins did not develop insulin resistance or diabetes [54]. These findings suggest that this inflammatory state caused by certain species of gut bacteria is contributing to obesity and insulin resistance. Also, Brugman et al. [56] have shown that by altering the gut microbiome in mice through the administration of antibiotics, there is a decreased incidence of developing type 1 diabetes and insulin resistance. Therefore, it can be concluded that a strong correlation exists between the gut microbiome and the inflammatory state seen in metabolic syndrome and insulin resistance by these interactions. However, additional studies are required to investigate whether there is a correlation between gut bacteria in influencing insulin receptor and GPCR protein activation.

#### 4.3.2. The role of Ghrelin on IR Signaling in Obesity

**Figure 5:**
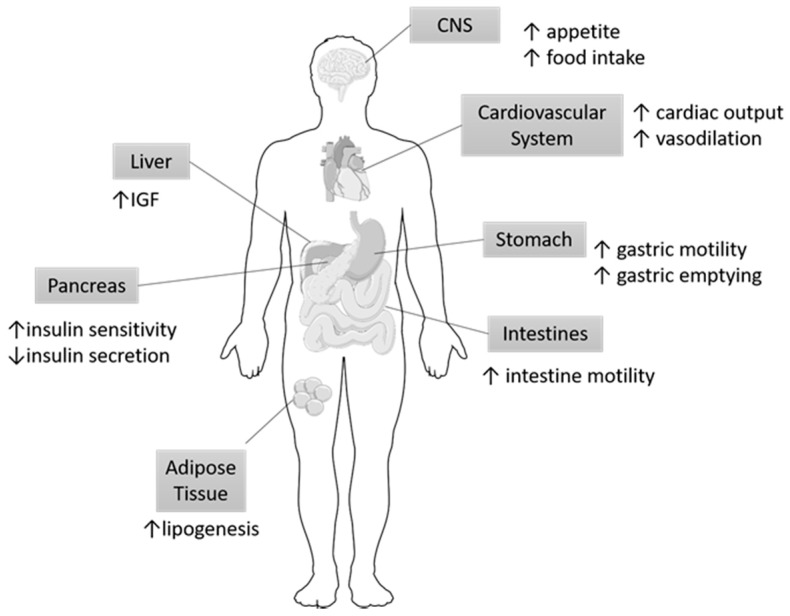
Organ specific targets of Ghrelin. Ghrelin has been shown to exert its action on multiple organs. Most notably, ghrelin modulates eating habits and has been correlated with metabolic syndrome. The arrows indicate the respective increase or decreases in specific functions of the organs participating in metabolism in response to Ghrelin. IGF, Insulin growth factor; CNS, central nervous system.

Ghrelin, a peptide hormone found primarily in the gastrointestinal tract, specifically in the oxytonic glands of the gastric fundus and the anterior pituitary gland, has been shown to play a role in metabolic disorders including obesity and T2DM [57,58]. Endogenous ghrelin stimulates the release of growth hormone (GH) and directly modulates feeding habits, glucose homeostasis, and energy balance [58]. Orexigenic neural circuitry maintains this systemic balance by communicating with the GI system and central nervous system (CNS) [59]. In addition to its complex role in providing energy, homeostasis, and GH secretion, alterations in ghrelin are also indicative of meal anticipation [60]. These mechanisms of action are illustrated in Figure 5 and depict the response of various organs of the GI tract to Ghrelin. These alterations are particularly significant because plasma ghrelin levels are inversely correlated with obesity, weight gain, and insulin resistance [61]. By extension, abnormally high levels of plasma ghrelin during states of hunger may be implicated in metabolic diseases and may be regulated through GPCRs and IR cross-talk mechanisms.

The proposed link between ghrelin and obesity was evaluated in a study by Tschöp, Smiley, and Heiman [58] that showed that ghrelin administration in mice and rats resulted in weight gain and decreased fat utilization. It was determined that the weight gain not be a direct result of hyperphagia but rather of an inadequate energy metabolism. Ghrelin injections during the resting phase and active feeding phase increased the respiratory quotient (RQ), indicating increased carbohydrate utilization and reduced fat utilization. Moreover, when the mice and rats were administered GH, there was an increase in energy utilization in both light and dark periods, indicating that GH acts independently from ghrelin in energy homeostasis.

Ghrelin exerts its action by binding to the ghrelin receptor (GHSR), which is also known as the growth hormone secretagogue receptor. The GHSR exists in two isoforms: 1a and 1b; however, 1a will be the primary focus of this paper. GHSR1a is a heterotrimeric GPCR known to stimulate growth hormone secretion when expressed on somatotrophs in the anterior pituitary gland. Ghrelin binding to GHSR1a results in a conformational change of the receptor and subsequent downstream intracellular signaling cascades [62]. Of particular interest is the signal transduction that occurs through calcium ion [Ca^2+^] signaling, as the downstream actions lead to an increase in cytosolic calcium [Ca^2+^]_i_ concentration in pancreatic β-islet cells that stimulate insulin release. These findings suggest that ghrelin stimulates insulin release and, furthermore, that Ca^2+^ may be a messenger signal for ghrelin in pancreatic β-cells [63]. Thus, ghrelin levels play a critical role in regulating insulin release and resistance.

More specifically, Murata et al. [64] reported that the activation of ghrelin/GHSR1a is involved in the alteration of IRS1 signaling. Physiologically, the IRS1 is activated by PI3K-AKT phosphorylation; however, ghrelin was shown to increase IRS1-associated PI3K-MAPK activity and inhibited AKT activity. This suggests that ghrelin up-regulates both tyrosine phosphorylation and insulin-dependent tyrosine phosphorylation of IRS1. Additionally, ghrelin and insulin showed an additive increase in the tyrosine phosphorylation of IRS1 [64]. This is particularly important as ghrelin and insulin-induced increases of PI3K and MAPK may be linked to an energy-rich state such as obesity, as these pathways regulate cell proliferation.

**Figure 6:**
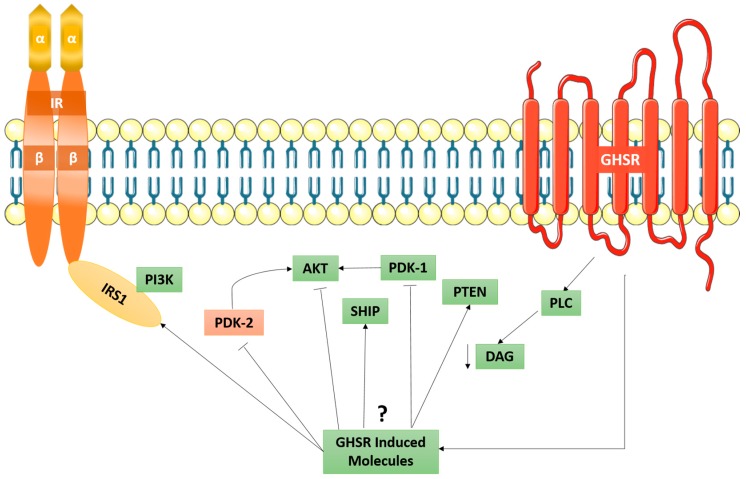
Proposed GHSR induced mechanism of signal transduction in relation to the IR. Here, the possible GHSR-induced molecules are shown to exert their effects by activating or inhibiting several signalling pathways. More importantly, it has been proposed that the inhibiting mechanism of AKT kinase by ghrelin is responsible for the disassociation of the IRS1-PI3K-AKT pathway. Taken in part from Murata et al. [64].

When ghrelin-induced IRS1 phosphorylation occurs, it does not occur through the IR, but rather through an independent cascade through the GHSR. Ghrelin does not increase the tyrosine phosphorylation of IRβ chain, as an antagonist for the GHSR resulted in a decrease in ghrelin-induced tyrosine phosphorylation of IRS1 [64]. As shown in Figure 6, it has been speculated that the ghrelin/GHSR downstream signaling cascade, which is a GPCR-pathway, can cross-talk with the insulin-signaling pathway through the IR. Collectively, the ghrelin/GHSR and the PI3K-AKT signaling pathway may suggest a role in the development of obesity. Furthermore, the observations made by Haxho et al. regarding NMBR-MMP9-IRβ crosstalk [8] may be applied to ghrelin in that it may initiate PI3K-AKT signaling via biased GPCR agonist-induced transactivation of IRβ.

In summary, ghrelin results in an overall anabolic state by increasing energy gain and GH secretion. Fasting and high plasma glucose levels increase the level of circulating ghrelin and may suggest an underlying link to metabolic diseases such as obesity. Though ghrelin is primarily released through the gastric fundus, it has been found in the hypothalamus, which suggests its role in energy homeostasis through the central nervous system [58,65]. Although the relationship between ghrelin and metabolic diseases has been investigated, it is possible that the upregulation of the GHSR signaling cascade with the primary agonist being ghrelin may have a cross-talk link between the neuroendocrine axis of GH and insulin signaling and the regulation of energy balance [58].

## 5. GPCR-IR Signaling in Pancreatic Cancer

Neuropeptides and their corresponding GPCRs have been implicated in the autocrine and paracrine stimulation of human cancers including pancreatic ductal adenocarcinoma (PDAC). The interactions with intracellular and transmembrane signal transduction pathways in relation to the insulin receptor, although not entirely understood, have been studied in-depth and shed some light on cancer cell progression, maturation, growth, and treatment [66,67,68]. Thus, the possibility of a link existing between GPCR-biased agonism and selectively moderating IR activation could have implications for the progression of pancreatic cancer.

In pancreatic cancer PANC-1 cells, crosstalk between GPCRs and IR receptor-signaling pathways has revealed that IR-dependent potentiation of mitogenic agonist, neurotensin, is related to the mammalian target of rapamycin complex 1 (mTORC1) pathway [69]. Following IR activation, the subsequent downstream signaling cascade results in the activation of AKT, which in turn activates mTORC1 [70]. Activation of mTORC1 regulates the PI3K-AKT pathway by negatively inhibiting IRS1 [71]. As previously outlined, this rapamycin-sensitive PI3K-AKT-mTORC1 signaling pathway is a key component of IR signaling in pancreatic cells [13]. The importance of the mTORC1 in regulating the IR has been further substantiated through studies investigating the effects of mTORC1 inhibition, as blocking the mTORC1 pathway causes inhibition of the IRS1 and potentiates the AKT pathway, which is followed by a subsequent increase in insulin sensitivity at the IR [71,72].

Concerning pancreatic cancer, studies have demonstrated that exposure to insulin results in a higher number of pancreatic cancer cells responding to low picomolar concentrations of neurotensin, a GPCR agonist [73]. This is of particular importance, as neurotensin stimulates tumor cell growth in addition to GPCR agonist neuropeptides gastrin-releasing peptide, neuromedin B, gastrin, cholecystokinin, and arginine vasopressin [74]. Interfering with this neuropeptide signaling, as well as intricate connections with other signaling pathways, might offer insight into new therapeutic approaches in the treatment of human cancers.

Therefore, as it relates to PDAC, the rapamycin-sensitive mTORC1 pathway mediates a link between IR and GPCR signaling pathways [75]. Blocking the mTORC1 pathway leads to abolition of the potentiation of GPCR and phosphatidylinositol 4,5-bisphosphate (PIP2)-inositol triphosphate (IP3)-induced calcium release. The release of calcium by IP3 and IP3-receptors in the endoplasmic reticulum is an important regulator of autophagy in cancers [76]. As such, a drug that blocks mTORC1 will have two-fold effect in patients with type 2 diabetes suffering from pancreatic ductal carcinoma [77,78]. Therefore, the potential crosstalk between IR and GPCR should be further studied in relation to the association of pancreatic cancer and diabetes because of this common pathway. Although the relationship between diabetes and pancreatic cancer has been investigated, the possibility of crosstalk between the IR and GPCRs will shed light on further pathogenesis and treatment of this deadly disease.

## 6. Polycystic Ovarian Syndrome (PCOS)

Polycystic Ovarian Syndrome (PCOS) is a commonly recognized endocrine and metabolic disease that affects women of reproductive age and can give rise to additional metabolic diseases, including T2DM [79]. While its etiology remains unknown, it is characterized by severe insulin resistance, hyperandrogenism, menstrual disorders, and polycystic ovaries that can be seen with ultrasound [80,81]. These abnormal levels of androgen, coupled with high concentrations of circulating insulin, inhibit sex hormone binding protein (SHBP) secretion and result in increased circulating androgen levels and exacerbated clinical symptoms [80].

PCOS is characterized by hyperinsulinemia and hyperandrogenism, the latter of which has been associated with an overexpression of ovarian RAS (OVRAS) [82]. Due to the routinely low estrogen and elevated androgen levels of PCOS follicles, the potential stimulatory action of ANG II on estrogen production has been suggested by several studies [83,84,85]. As outlined in Section 4.1, ANG II is the predominant player within the RAS system and contributes to insulin resistance. The relationship between ANGII, adipogenesis, and insulin resistance has been comprehensively examined elsewhere [86,87,88]. In brief, these relationships demonstrated that angiotensinogen and ACE were found in adipocytes in obese humans, thereby raising the possibility that ANG II was produced locally and could exert local effects contributing to insulin resistance [89]. Moreover, culturing of granulosa cells exhibited upregulation of ANG II receptors (angiotensin type 1 receptor, AT_1_R, and angiotensin type 2 receptors, AT_2_R) [86]. Intrinsic generation of angiotensin by ovarian follicles may have regulatory feedback mechanisms in OVRAS components such as angiotensinogen, renin, and ANG II receptors [90].

An additional contributing factor, suggested by Dunaif, is the possibility of a defect in the primary steps of insulin signaling, which could result in insulin resistance in women with PCOS [91]. This report observed that 50% of PCOS-positive fibroblasts (PCOS-ser) displayed a marked decrease in autophosphorylation of the IR [91]. Subsequent phosphor-amino acid analysis showed decreased insulin-dependent receptor tyrosine phosphorylation and increased insulin-independent receptor serine phosphorylation. The remaining 50% of fibroblasts in PCOS-positive women (PCOS-nl) presented with no aberrations in phosphorylation of the IR, and the phenotype and degree of insulin resistance were identical to that of PCOS-ser fibroblasts [91]. These findings suggest that a defect downstream of IR binding to its ligand, such as phosphorylation of IRS1 or activation of PI3K, is responsible for insulin resistance in the subset of PCOS-nl women.

Therefore, if a post-binding defect in receptor signaling is due to increased phosphorylation of the IR and IRS1, it may be the case that upregulation of a GPCR-biased agonist is stimulating said phosphorylation. The most likely candidate for the upregulation of a GPCR agonist may be angiotensin [8]. As mentioned previously, ANG II is a potent GPCR agonist that induces phosphorylation of the IRβ and IRS1, and the increased number of ANG II receptors that exist in granulosa cells of the ovary suggests a critical link that may exist between the IR and GPCRs.

## 7. GPCR-IR Signaling Cross-Talk: Potential Implications in Alzheimer’s Disease and Neurocognition

**Figure 7:**
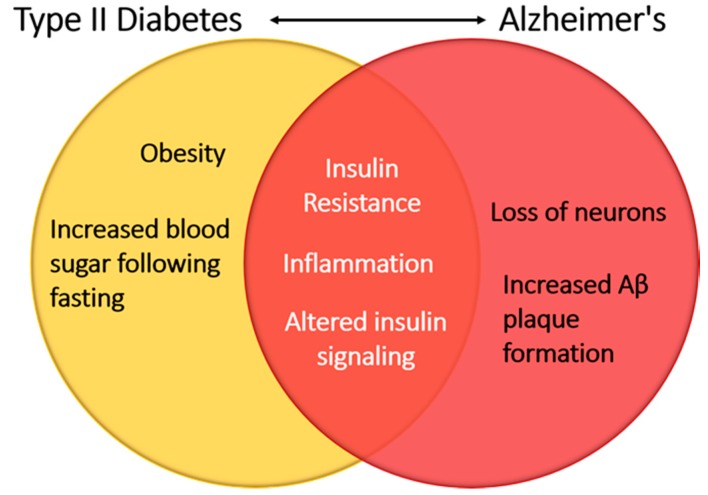
Insulin signaling linking Type II diabetes and Alzheimer’s disease. Alterations in glucose homeostasis have led to modification of insulin receptor signaling. Within the central nervous system, insulin receptor signaling perturbations result in the hallmarks of neurodegenerative diseases such as increased β-amyloid plaque formation and a loss of neurons.

There is a growing body of evidence that metabolic syndrome and insulin resistance can have an effect on the brain and cause neurocognitive impairment and dementia, with the most notable causes being micro- and macrovascular changes throughout the body [92,93,94]. In healthy individuals, insulin plays a neuroprotective role in the brain and can help prevent apoptosis of neurons, β amyloid toxicity, oxidative stress, and ischemia [95]. However, patients with type I or type II diabetes mellitus have been shown to suffer from cognitive regression [96]. These findings suggest that alterations in glucose homeostasis are a hallmark of these diseases and perturbations in insulin signaling may contribute to the neurocognitive deficits seen in these patients (Figure 7).

Insulin receptors are dispersed throughout the brain but are more pronounced in the hippocampus, olfactory bulb, hypothalamus, amygdala, substantia nigra, basal ganglia, and frontal cortex [97]. These receptors have been implicated in cognitive brain functions such as learning and memory within rodent hippocampus and cerebral cortex, respectively [98,99]. When insulin function becomes impaired as a result of conditions such as diabetes, insulin resistance may develop. Consequently, the sustained high level of insulin within the periphery decreases the sensitivity of receptors to glucose uptake [100]. Peripheral insulin resistance has been shown to contribute to insulin resistance in the brain by reducing available insulin levels and increasing the levels of beta amyloid (Aβ) formation [101]. This effect can lead to neuronal cell death as Aβ deposition can cause dysfunction in lipid metabolism, signaling cascades, autophagy regulation, and neurotransmitter dysfunction [102]. Two sources of insulin are available to the brain: peripheral insulin, which is heavily correlated with T2DM [103], and central insulin. Peripheral insulin must cross the blood-brain barrier (BBB), which contains brain endothelial cells that have insulin receptors, and is in communication with both the extravascular and CNS circulations [104]. Peripheral insulin is taken up by receptor-mediated transport across endothelial cells of the BBB directly, as well as via receptors expressed on the endothelial cells of the BBB via induction of a secondary signaling cascade [105]. Direct entry of insulin via receptor-mediated transport has been shown to interact with insulin receptors expressed in the brain, thereby influencing the brain’s physiology [95].

The recent progress in the treatment of diabetes has depended on a continuously evolving understanding of GPCRs and their potential for innovation and drug discovery [106]. The study of GPCRs in the CNS has also led to a deeper appreciation of their role in the development of Alzheimer’s disease and involvement in cleavage of the amyloid precursor protein via α-, β-, and γ-secretase [107]. Alzheimer’s is a neurodegenerative disorder that causes cognitive impairment and memory deficits that lead to dementia due to tau protein hyperphosphorylation and amyloid plaque accumulation [108]. Of particular interest is the 5-hydroxytryptamine 2C receptor (5-HT_2c_) receptor within the choroid plexus, as accumulating evidence suggests that this GPCR may be linked to insulin within the CNS [109]. In rodent models, insulin and IGF-1 signaling were shown to downregulate 5-HT_2c_ receptor function in the choroid plexus [109]. Since the 5-HT_2c_ receptor has been proposed to regulate cerebral spinal fluid (CSF) production, and due to insulin having an inhibitory effect on 5-HT_2c_ receptor activity, insulin may increase CSF production and change the composition and volume of the CSF [109]. These findings are further confirmed by the co-localization of the 5-HT_2c_ receptor and IRs within different parts of the brain [110,111].

Although more research is required to support the existence of a link between GPCR-biased agonism as it related to the IR, theoretically, targeting a GPCR connected to IRs within the CNS may be a possible avenue to develop a more potent therapy to prevent Alzheimer’s disease. Both insulin and the IR have also been suggested to have protective properties against beta-amyloid-induced cell death, with one study citing insulin as an inhibitor of beta-amyloid fibrillation networks [112]. It is, therefore, worthwhile to better understand how we can utilize GCPR in relation to the insulin receptor and its relationship with metabolic disease, diabetes, and increased insulin resistance.

## 8. Conclusions

The insulin receptor is expressed in a diverse range of cells throughout the body, and thus aberrations in its activation patterns can directly or indirectly lead to several pathologies. One such disease state is a metabolic syndrome, a collection of pathologies related to metabolism in addition to other conditions such as PCOS, Alzheimer’s, and cancer. The activity of the insulin receptor has been shown to be regulated by the GPCRs through the novel phenomenon of GPCR biased agonism. This concept is a particularly vital avenue to continue to explore, as the potential for GPCR biased agonism to affect the insulin receptor may perhaps shed light on the underlying mechanisms involved in contributing to or exacerbating disease conditions related to the insulin receptor. 

## Abbreviations

ACE	Angiotensin Converting Enzyme
AKT	Protein kinase B
ANGII	Angiotensin II
AT_1_R	Angiotensin type 1 receptors
Aβ	Beta amyloid
B_1_R	Bradykinin Type I Receptor
B_2_R	Bradykinin Type II Receptor
BBB	Blood Brain barrier
BK	Bradykinin
CNS	Central Nervous System
CSF	Cerebrospinal Fluid
DAG	Diacylglycerol
DDS	Dextran sulfate sodium
EBP	Elastin binding protein
ERK	Extracellular signal-regulated kinase
GH	Growth hormone
GHSR	Ghrelin receptor
GI	Gastrointestinal
GPCR	G-protein coupled receptor
IBD	Irritable Bowel Syndrome
IP_3_	Inositol-1,4,5-triphosphate
IR	Insulin receptor
IRS1	Insulin receptor substrate 1
JNK	c-Jun N-terminal kinase
KKS	Kallikrein-kinin System
LPS	Lipopolysaccharides
MetSyn	Metabolic Syndrome
MLCK	Myosin light chain kinase
MMP9	Matrixmetalloproteinase-9
mTORC1	Mammalian target of rapamycin complex 1
Neu-1	Neuraminidase-1
NMBR	Neuromedin B receptor
NO	Nitric oxide
OVRAS	Ovarian Renin Angiotensin System
PCOS	Polycystic Ovarian Syndrome
PDAC	Pancreatic ductal adenocarcinoma
PI3K	Phosphatidylinositol 3-kinase
PIP2-IP3	4,5-bisphosphate-Inositol triphosphate
PKC	Protein kinase C
PLC	Phospholipase C
RAS	Renin Angiotensin System
RQ	Respiratory quotient
RTK	Receptor tyrosine kinases
SCFA	Short chain fatty acids
SHBP	Sex hormone binding protein
T2DM	Type two diabetes mellitus
UC	Ulcerative colitis
